# Ischemia-induced ACSL4 activation contributes to ferroptosis-mediated tissue injury in intestinal ischemia/reperfusion

**DOI:** 10.1038/s41418-019-0299-4

**Published:** 2019-02-08

**Authors:** Yang Li, Dongcheng Feng, Zhanyu Wang, Yan Zhao, Ruimin Sun, Donghai Tian, Deshun Liu, Feng Zhang, Shili Ning, Jihong Yao, Xiaofeng Tian

**Affiliations:** 1 Department of General Surgery, The Second Affiliated Hospital of Dalian Medical University, Dalian, 116023 China; 2 Department of Pharmacology, Dalian Medical University, Dalian, 116044 China

**Keywords:** Cell biology, Gastrointestinal diseases

## Abstract

Ferroptosis is a recently identified form of regulated cell death defined by the iron-dependent accumulation of lipid reactive oxygen species. Ferroptosis has been studied in various diseases such as cancer, Parkinson’s disease, and stroke. However, the exact function and mechanism of ferroptosis in ischemia/reperfusion (I/R) injury, especially in the intestine, remains unknown. Considering the unique conditions required for ferroptosis, we hypothesize that ischemia promotes ferroptosis immediately after intestinal reperfusion. In contrast to conventional strategies employed in I/R studies, we focused on the ischemic phase. Here we verified ferroptosis by assessing proferroptotic changes after ischemia along with protein and lipid peroxidation levels during reperfusion. The inhibition of ferroptosis by liproxstatin-1 ameliorated I/R-induced intestinal injury. Acyl-CoA synthetase long-chain family member 4 (ACSL4), which is a key enzyme that regulates lipid composition, has been shown to contribute to the execution of ferroptosis, but its role in I/R needs clarification. In the present study, we used rosiglitazone (ROSI) and siRNA to inhibit ischemia/hypoxia-induced ACSL4 in vivo and in vitro. The results demonstrated that ACSL4 inhibition before reperfusion protected against ferroptosis and cell death. Further investigation revealed that special protein 1 (Sp1) was a crucial transcription factor that increased ACSL4 transcription by binding to the ACSL4 promoter region. Collectively, this study demonstrates that ferroptosis is closely associated with intestinal I/R injury, and that ACSL4 has a critical role in this lethal process. Sp1 is an important factor in promoting ACSL4 expression. These results suggest a unique and effective mechanistic approach for intestinal I/R injury prevention and treatment.

## Introduction

Intestinal ischemia/reperfusion (I/R) injury, which is associated with a high mortality rate, occurs in numerous clinical pathologies such as small intestinal volvulus, acute mesenteric ischemia, shock, and trauma. Intestinal I/R injury can be induced by some surgical approaches such as small bowel transplantation [1–3]. Massive epithelial cell death is a major cause of intestinal mucosal barrier dysfunction, which leads to systemic inflammation and dysfunction of remote organs [4]. Recently, I/R injury events have been increasingly shown to involve nonapoptotic pathways such as necroptosis [5, 6], pyroptosis [7, 8], and ferroptosis [9, 10], which has attracted substantial attention and intense interest.

Ferroptosis, a recently recognized form of regulated cell death (RCD), is identified as iron-dependent and caspase-independent nonapoptotic cell death [11]. Ferroptosis differs from other classical nonapoptotic cell death programs by its characteristics of mitochondrial shrinkage and increased mitochondrial membrane density (morphological), iron and lipid reactive oxygen species (L-ROS) accumulation (biochemical), and involvement of a unique set of genes (genetic) [12, 13]. The biochemical mechanism underlying ferroptosis is the iron-catalyzed formation of lipid radicals combined with the depletion of glutathione (GSH) or the inactivation of the lipid repair enzyme GSH peroxidase 4 (GPx4) [14, 15]. Lipid peroxidation is essential for ferroptosis and involves the preferential oxidation of arachidonic acid (AA) and its esterifiable production phosphatidylethanolamine (PE) [16–18]. Both are types of abundant polyunsaturated fatty acids (PUFAs) in the intestinal epithelium [19, 20]. PUFAs can be oxidized into metabolites (e.g., hydroxyeicosatetraenoic acids (HETEs)) during the ferroptosis process [9, 21–23]. Treatment with L-ROS inhibitors such as liproxstatin-1 can suppress this lethal process [9, 24, 25].

Ferroptosis has been implicated in several pathophysiological contexts such as degenerative diseases, tumor suppression, antiviral immunity, and stroke [12, 13]. Recent studies indicate that liproxstatin-1 alleviates I/R-induced liver injury in mice [9], and that deferoxamine, compound 968, and ferrostatin-1, all of which inhibit glutaminolysis and ferroptosis, limit myocardial I/R injury ex vivo [10]. However, the relevance of ferroptosis in intestinal I/R is still unexplored and remains enigmatic.

Conventional I/R studies have focused on the reperfusion phase and are of limited translational value. However, although it is part of this dynamic process, the ischemic phase is often overlooked [26]. Chouchani et al. [27] have reported that succinate accumulation during ischemia contributes to reperfusion injury in multiple organs; in contrast with the traditional outlook, this study focused on the ischemic phase. This study not only provided new insight into I/R but also indicated a potential avenue for effective clinical interventions. These findings prompted us to explore the possibility of a certain core regulator induced by ischemia that could lead to ferroptosis immediately after reperfusion in the intestine.

Acyl-CoA synthetase long-chain family member 4 (ACSL4) has been studied as a crucial factor in metabolism-associated diseases [28]. ACSL4 was recently found to facilitate the esterification of arachidonoyl (AA) and adrenoyl into PE, a process closely related to ferroptosis [21, 22]. Suppression of this process by genetic and pharmacological ACSL4 inhibition triggers an antiferroptotic rescue pathway [21]. Thus, ACSL4 has been identified as not only a sensitive regulator of ferroptosis but also an important contributor to ferroptosis [29]. To date, ACSL4 has rarely been reported in I/R-related diseases. Hence, the functional role and significance of ACSL4 and its correlation with ferroptosis need to be deeply understood in the context of intestinal I/R injury.

Special protein 1 (Sp1) is a classical and well-studied transcription factor that can activate target genes by binding to GC-box elements in promoter regions [30–32]. Previous studies reported that hypoxia induced Sp1 expression in the nucleus and led to cell injury [33, 34]. As predicted, the *ACSL4* gene contains many GC boxes and could be a potential target of Sp1. However, the correlation between Sp1 and ACSL4 in I/R injury associated with ferroptosis remains unknown.

In this study, we hypothesize that ferroptosis participates in intestinal I/R injury, and that the activation of ACSL4 during ischemia contributes to ferroptosis after reperfusion. Our findings demonstrate that the inhibition of ferroptosis is a critical mechanism protecting against intestinal I/R injury. Inhibition of ACSL4 rescues the tissue injury caused by I/R-mediated ferroptosis in the intestine. We aim to provide a new viewpoint and target in the treatment of intestinal I/R injury.

## Materials and methods

### Murine model of intestinal ischemia and I/R

Male C57BL/6 mice (8 weeks old, specific pathogen-free) were purchased from the Animal Center of Dalian Medical University (Dalian, China). The mice were fed standard food and water, and were acclimated to the environment before use. All mice were anesthetized by an intraperitoneal (i.p.) injection of pentobarbital (50 mg/kg body weight). To establish the ischemia model, a midline laparotomy was performed and the superior mesenteric artery was occluded by a microvascular clamp for 30, 45, or 60 min without reperfusion. To establish the I/R model, the clamp was removed after 45 min of occlusion and the blood supply was recovered for various durations as required for the present study. All procedures were conducted according to the Institutional Animal Care Guidelines and were approved by the Institutional Ethics Committee of Dalian Medical University.

### Cell culture, hypoxia, and hypoxia/reoxygenation procedures

Caco-2 cells were purchased from the American Type Culture Collection (HTB-37, USA) and cultured in a humidified incubator maintained at 37 °C and 5% CO_2_ in Dulbecco’s modified Eagle’s medium (11965, Gibco, MD, USA) containing 10% fetal bovine serum (0500, ScienCell, CA, USA), 1% non-essential amino acids (M7145, Sigma, MO, USA) and 1% glutamine (G3126, Sigma). The cell line was authenticated with short tandem repeat profiling. To establish the hypoxia model, cells were incubated in a microaerophilic system (Thermo, WA, USA) with 5% CO_2_ and 1% O_2_ balanced with 94% N_2_ gas for 12 h. For reoxygenation, cells were cultured under normoxic conditions for 2 h.

### Inhibitor treatment and serum assay

Liproxstatin-1 (S7699, Selleck, TX, USA), a ferroptosis inhibitor, was administered i.p. at a concentration of 10 mg/kg 1 h before ischemia induction, in accordance with previous study protocols [9]. Mice were killed at 30 min of reperfusion and serum was collected from the abdominal aorta. In addition, liproxstatin-1 dissolved to a final concentration of 200 nM was used to treat Caco-2 cells in vitro for 12 h before hypoxia induction. Rosiglitazone (ROSI, S2556, Selleck), a classic peroxisome proliferator-activated receptor-γ agonist that has been used for ACSL4 inhibition, was administered intravenously at a concentration of 0.4 mg/kg 1 h before ischemia induction, as pretreatment of ROSI allows sufficient time for proper phospholipid remodeling in the membranes [21, 22]. Mice were killed at 45 min of ischemia or at 30 min of reperfusion. Serum was collected from the abdominal aorta. Kits were used to assay the levels of tumor necrosis factor (TNF)-α (ab208348, Abcam, MA, USA), interleukin (IL)-6 (ab100712, Abcam), and ACSL4 activity (ab241005, Abcam).

### Cell viability and lactate dehydrogenase assay

Cell viability was tested by a Cell Counting Kit-8 (CCK-8, CK04, Dojindo, Tokyo, Japan) assay. In brief, the reagent, which was diluted to the working concentration, was added to a 96-well plate and incubated at 37 °C for 2 h. Optical density (OD) values were measured at 450 nm by a Thermo Multiskan FC microplate photometer.

Serum and cellular supernatants were collected for the lactate dehydrogenase (LDH) assay (WLA072b, Wanleibio, Liaoning, China) according to the manufacturer’s instructions. OD values were measured at 450 nm by a microplate photometer.

### Iron measurements

Fresh ischemic intestines were immediately homogenized with phosphate-buffered saline (PBS). The supernatant was collected after centrifugation. The iron level was determined by the Iron Assay Kit (ab83366, Abcam) according to the manufacturer’s instructions.

### Transmission electron microscopy

After reperfusion, the mice were killed and the intestines were washed with precooled PBS (pH 7.4). Part of the intestine was then removed and incubated overnight in 0.1 M PBS (pH 7.4) containing 2.5% glutaraldehyde. The target tissues were cut into 50 µm-thick sections using a vibratome. Selected areas of the intestines were postfixed in 1% osmium tetroxide for 1 h, dehydrated in a graded ethanol series, and embedded in epoxy resin. Polymerization was performed at 80 °C for 24 h. Ultrathin sections (100 nm) were cut, stained with uranyl acetate and lead citrate, and viewed under a JEM2000EX transmission electron microscope (TEM; JEOL, Tokyo, Japan). We randomly chose five fields for each sample and counted the mitochondria with ferroptotic features [12]. The number of ferroptotic mitochondria per field in each sample was quantified.

### Western blotting

Tissues and cells were lysed in total protein lysis buffer (P0013, Beyotime, Shanghai, China). The supernatant was quantified by a BCA kit (DQ111-01, TransGen, Beijing, China). Western blotting was performed with the following primary antibodies: ACSL4 (ab155282, Abcam), GPx4 (ab125066, Abcam), FTH1 (ab65080, Abcam), COX2 (ab62331, Abcam), and β-actin (TA-09, ZSGB-BIO, Beijing, China).

Nuclear protein was extracted using the ProteinExt Mammalian Nuclear and Cytoplasmic Protein Extraction Kit (DE201-01, TransGen, Beijing, China) according to the manufacturer’s instructions. Sp1 (PA5-29165, Invitrogen, WA, USA) and Lamin B1 (12987-1-AP, Proteintech, Hubei, China) primary antibodies were used for western blotting.

### Transient transfection

All plasmids and small interfering RNAs (siRNAs) were created and synthesized by GenePharma (Shanghai, China). Before transfection, cells were transplanted to 6-well or 96-well plates and grown to a confluence of 40~50%. Lipofectamine^TM^ 3000 (L3000015, Invitrogen) was used according to the manufacturer’s protocol. The Sp1 plasmid was subcloned into the pEX-4 vector to overexpress Sp1. The following siRNA sequences were used: ACSL4 sense 5′-GAGGCUUCCUAUCUGAUUATT-3′ and anti-sense 5′-UAAUCAGAUAGGAAGCCUCTT-3′; Sp1 sense 5′-CUGGUCAAAUACAGAUCAUTT-3′ and anti-sense 5′-AUGAUCUGUAUUUGACCAGTT-3′; and negative control sense 5′-UUCUCCGAACGUGUCACGUTT-3′ and anti-sense 5′-ACGUGACACGUUCGGAGAATT-3′. All siRNAs and plasmids were incubated with the transfection media for 2 days before hypoxia/reoxygenation (H/R).

### Lipid peroxidation assay

A lipid peroxidation assay kit (A106, Jiancheng, Jiangsu, China) was used to test the lipid peroxidase (LPO) level in lysates of intestines and cells following the manufacturer’s protocol. Briefly, lipid peroxide reacts with chromogenic reagents under the condition of 45 °C for 60 min and produces a stable chromophore with a maximum absorption peak at 586 nm. Lipid ROS was measured by using the live-cell analysis reagent BODIPY 581/591 C11 (D3861, Invitrogen). Transplanted cells were incubated with the kit reagent at a working concentration of 2.5 µM for 30 min in an incubator. Images were acquired under an IX83 fluorescence microscope (Olympus, Tokyo, Japan).

### GPx4 activity assay

GPx4 activity was assayed by using phosphatidylcholine hydroperoxide as a substrate, following the previously described protocol [35].

### Myeloperoxidase and GSH assays

Myeloperoxidase (MPO) levels in the lung and liver were detected by an MPO assay kit (A044, Jiancheng).

Ischemic intestines were homogenized and the supernatant was collected for GSH analysis using a total GSH/oxidized GSH assay kit (A06, Jiancheng).

### Intestinal permeability assay

Changes in intestinal barrier permeability after I/R were measured by 4.4 kDa fluorescein isothiocyanate-dextran (FD-4, Sigma-Aldrich, USA) in serum. Two hundred microliters of PBS containing 25 mg/ml FD-4 was given by gavage during ischemia, as reported in a previous study [36]. Blood was collected from the abdominal aorta after reperfusion and then centrifuged to separate the serum. The fluorescence intensity of each sample (100 μl serum) was measured with the Varioskan LUX Multimode microplate reader (Thermo Scientific, USA; excitation, 480 nm; emission, 520 nm).

### Transepithelial electrical resistance assay

To assess transepithelial electrical resistance (TEER) after H/R in vitro, Caco-2 cells were seeded in the upper chamber of the transwell plate system (0.4 µm pore size, Costar Incorporated Corning, NY) at a concentration of 1 × 10^5^ cells/ml. Caco-2 cells were treated with liproxstatin-1 or transfected with siRNA, as described above. Then, the resistance was measured by using Millicell-ERS device (Millipore, MA, USA). TEER was calculated by the following equation: TEER = (R1 − R0) × A Ω cm^2^; here, R1 is the resistance of filter with cells, R0 is the resistance of filter alone, and A is the area of filter.

### Tissue histology and immunofluorescence

For the histological analysis, intestinal tissues were fixed in 4% formalin and then paraffin-embedded. Four-micrometer-thick sections were stained with hematoxylin and eosin (H&E).

Caco-2 cells were fixed in 4% paraformaldehyde for 1 h, washed three times with PBS, permeabilized with 0.1% Triton X-100 for 15 min, and blocked with 2% bovine serum albumin in PBS at 37 °C for 1 h. The cells were incubated with the Sp1 primary antibody at 4 °C overnight, washed three times with PBS, and incubated with Alexa Fluor 594-conjugated secondary antibody (SA00006-4, Proteintech) at 37 °C for 1 h. After additional PBS washes, the cells were counterstained with the nuclear stain 4,6-diamidino-2-phenylindole (C1006, Beyotime) at room temperature for 10 min. A laser scanning confocal microscope (TCS SP5II, Leica, Wetzlar, Germany) was used to acquire immunofluorescence images, quantified by ImageJ (NIH, USA).

### 5-HETE assay

5-HETE, an ACSL4-mediated product of AA oxidation [29], in lysates of intestines and cells was assessed using a 5-HETE ELISA kit (CED739Ge, Uscnlife, Hubei, China) according to the manufacturer’s instructions.

### 12/15-HETE assay

12/15-HETE levels were assessed using 12/15-HETE ELISA kits (ab133034/ab133035, Abcam) according to the manufacturer’s instructions.

### Real-time PCR

Total RNA was isolated from intestines and Caco-2 cells with RNAiso Plus (9109, Takara, Liaoning, China). ACSL4 mRNA was reverse transcribed to cDNA with the PrimerScript^TM^ RT Reagent Kit (RR047A, Takara), according to the manufacturer’s instructions. ACSL4 mRNA expression was quantified using TB Green^TM^ Premix Ex Taq^TM^ II (RR820A, Takara). ACSL4 mRNA levels were normalized to β-actin mRNA levels. The following specific primers were synthesized by Invitrogen: human ACSL4 primers, forward 5′-TCTGCTTCTGCTGCCCAATT-3′ and reverse 5′-CGCCTTCTTGCCAGTCTTTT-3′; human β-actin primers, forward 5′-GGGAAATCGTGCGTGACATT-3′ and reverse 5′-GGAACCGCTCATTGCCAAT-3’; mouse ACSL4 primers, forward 5’-CCACACTTATGGCCGCTGTT-3′ and reverse 5′-GGGCGTCATAGCCTTTCTTG-3′; and mouse β-actin primers, forward 5′-ACTGCCGCATCCTCTTCCT-3′ and reverse 5′-TCAACGTCACACTTCATGATGGA-3′.

### Chromatin immunoprecipitation assay

Purification of sonicated nuclear lysates and immunoprecipitation were performed using a chromatin immunoprecipitation (ChIP) assay kit (P2078, Beyotime) according to the manufacturer’s instructions.

### Dual-luciferase reporter assay

Wild-type (WT) and mutant human ACSL4 promoters were synthesized and subcloned into the pGL3-Basic vector by GenePharma (Shanghai, China). An empty vector (control) or the WT, or deleted (Del) ACSL4 promoter plasmid (1.5 μg/well) was transfected into Caco-2 cells. Then, cells were lysed and analyzed with the TransDetect Double-Luciferase Reporter Assay Kit (FR201, TransGen). Luciferase activities were measured using a Varioskan LUX multimode microplate reader (Thermo Scientific, USA).

### Acquisition of clinical samples

The collection and use of human intestinal specimens were approved by the local ethics committee of Dalian Medical University. Patient intestinal samples were obtained after the patients provided informed written consent. Ischemic intestinal segments, which were designated the model group, and the margins of the resected intestinal segments, which were designated the normal group, were obtained from patients who underwent surgery for acute superior mesenteric artery embolism or strangulated intestinal hernia in the Department of Acute Abdominal Surgery of the Second Affiliated Hospital of Dalian Medical University. The intestinal samples were immediately frozen in liquid nitrogen and stored at − 80 °C until analysis.

### Statistical analysis

All values are expressed as the mean ± SD. Data with normal distributions were compared using one-way analysis of variance followed by the Student−Newman–Keuls test. The survival study results were analyzed using the Kaplan–Meier method. A two-tailed Student’s *t*-test was used to compare means between two groups. At least three independent experiments were performed to confirm the results. Statistical analysis was performed using GraphPad Prism 5.0 (GraphPad Prism Software, CA, USA). A *p*-value < 0.05 was considered statistically significant.

## Results

### Ferroptosis is present at the early stage of reperfusion

**Fig. 1:**
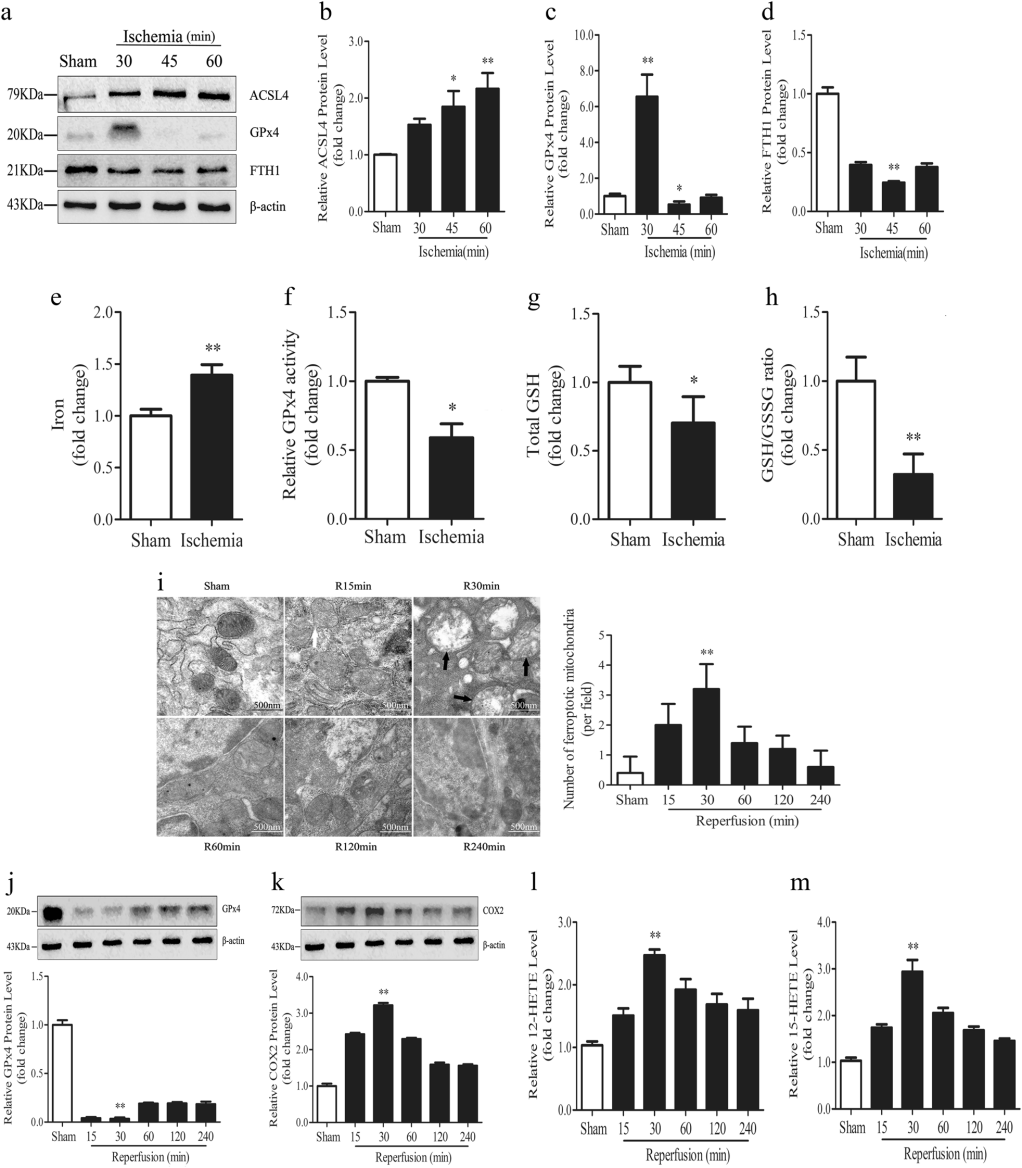
Ferroptosis is present in intestinal I/R injury. **a**–**d** Mice were subjected to intestinal ischemia (I) or sham surgery (sham) and divided into four groups: sham, I30min, I45min, and I60min (*n* = 6). Intestines were collected after ischemia. The expressions of ACSL4, GPx4, and FTH1 were detected by western blotting (*n* = 3). **e**–**h** Mice were subjected to 45 min of intestinal ischemia and intestines were collected to measure iron, GPx4 activity, GSH level, and the GSH/GSSG ratio after ischemia (*n* = 6). **i**–**m** Mice were subjected to 45 min of ischemia and different durations of reperfusion (R15min, R30min, R60min, R120min, and R240min). All samples were collected at the respective reperfusion times. **i** Representative TEM images and quantification are shown. The white arrow indicates outer mitochondrial membrane rupture and the black arrows indicate the reduction or disappearance of mitochondrial cristae. **j**, **k** GPx4 and COX2 protein levels in intestines were analyzed by western blotting (*n* = 3). **l**, **m** 12-HETE and 15-HETE levels were assayed by ELISA kits (*n* = 6). All results are expressed as the mean ± SD. *p < 0.05, **p < 0.01 vs. the sham group

Some core factors, such as FTH1, ACSL4, and GPx4, are accepted as pivotal and valid proteins in ferroptosis regulation [12, 14]. Therefore, to evaluate ferroptosis sensitivity after ischemia in the intestine, we determined the expression levels of these proteins under ischemic conditions. As shown in Fig. 1a-d, the expression of the positive regulator ACSL4 was induced in ischemic intestines compared with that in normal intestines. Meanwhile, the levels of two negative regulators (GPx4 and FTH1) were reduced after 45 min of ischemia. In addition, we assayed iron, GPx4 activity, GSH level, and GSH/GSSG ratio at 45 min ischemia in the intestine. Similar to the above results, iron, another essential factor for ferroptosis execution, showed significant cytoplasmic accumulation in the ischemia group but not in the sham group (Fig. 1e), and ischemia led to a reduction of GPx4 activity, GSH level, and GSH/GSSG ratio (Fig. 1f-h). These results suggest that ischemia induced changes in ferroptosis-related factors, which may sensitize the intestine to ferroptosis during subsequent oxygen replenishment by reperfusion.

Previous studies have shown that ferroptosis is involved in I/R injury in the liver [37], brain [38], kidney [9], and heart [10]. In addition, the results above indicated that reperfusion was likely to lead to ferroptosis after 45 min of ischemia. Therefore, to detect ferroptosis after reperfusion, we established an I/R model with various durations of reperfusion. TEM was used to investigate the morphological features of ferroptosis. The results showed that 15 min of reperfusion resulted in rupture of the outer mitochondrial membrane (Fig. 1i, white arrow), and that the disappearance of mitochondrial cristae was more prominent at 30 min (Fig. 1i, black arrow). However, similar features were not observed in prolonged reperfusion durations (Fig. 1i). In addition, the expression of GPx4 was decreased at 30 min of reperfusion (Fig. 1j), whereas the expression of COX2 was increased (Fig. 1k). 12-HETE and 15-HETE were also found to have a significant increase at 30 min of reperfusion (Fig. 1l, m). The results showed that ferroptosis was more active at 30 min than at other times after reperfusion, consistent with the TEM results. These findings indicate that reperfusion induces ferroptosis at the early stage, suggesting that ferroptosis may be involved in intestinal I/R injury.

### Inhibition of ferroptosis ameliorates I/R-induced intestinal injury

**Fig. 2:**
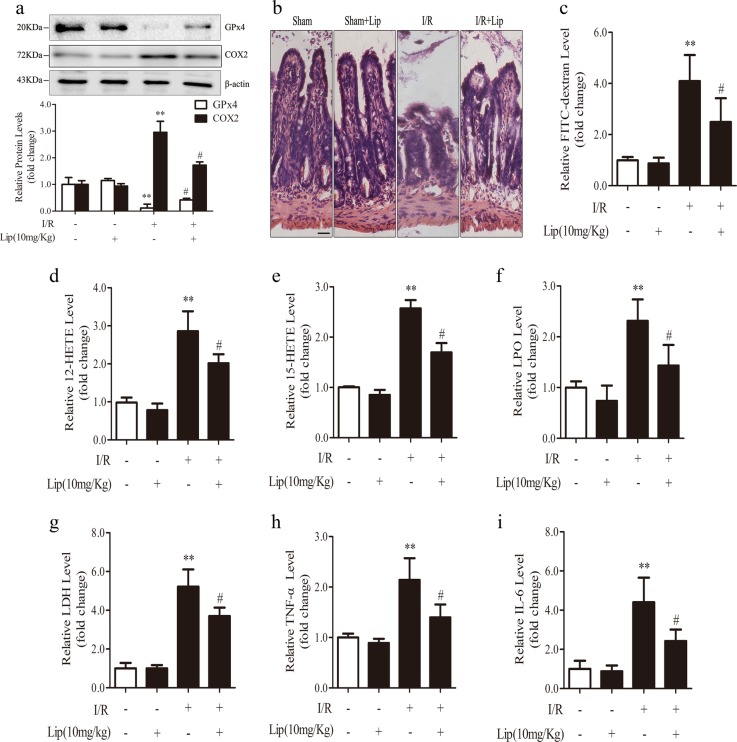
Liproxstatin-1 ameliorates ferroptosis and intestinal injury in vivo. Mice were treated with liproxstatin-1 (10 mg/kg) by intraperitoneal injection 1 h before ischemia and then subjected to ischemia/reperfusion (I, 45 min of ischemia; R, 30 min of reperfusion) or sham surgery (sham). All samples were collected after I/R. **a** GPx4 and COX2 levels were assessed by western blotting (*n* = 3). **b** Representative H&E-stained intestinal slices were imaged by microscopy (scale bar = 100μm). **c** The intestinal permeability was detected by measuring serum FD-4 content (*n* = 6). **d**–**f** 12-HETE, 15-HETE, and LPO were assayed by corresponding kits (*n* = 6). **g**–**i** Serum from mice was used to detect the levels of LDH, TNF-α, and IL-6 (*n* = 6). All results are expressed as the mean ± SD. **p < 0.01 vs. the sham group; #*p* < 0.05 vs. the I/R group

To further verify the existence of ferroptosis and its role in intestinal I*/*R injury, we determined whether the inhibition of ferroptosis could rescue intestinal injury. Liproxstatin-1, a potent and specific ferroptosis inhibitor that was previously shown to attenuate I/R damage [9, 25], was administered to mice, to determine the role of ferroptosis inhibition in intestinal I/R injury. In vivo, liproxstatin-1 treatment (i.p., 1 h before ischemia) induced GPx4 expression and reduced COX2 expression (Fig. 2a). Ferroptosis inhibition by liproxstatin-1 greatly attenuated intestinal villus damage and intestinal permeability as evidenced by H&E staining and serum FD-4 content, respectively (Fig. 2b, c). Furthermore, liproxstatin-1 decreased lipid peroxidation as indicated by the decrease in 12-HETE, 15-HETE, and LPO levels (Fig. 2d-f). Liproxstatin-1 also reduced serum LDH, TNF-α, and IL-6 levels (Fig. 2g-i). In addition, we found that orally administered liproxstatin-1 alleviated intestinal I/R injury (Fig. S1A, B).

**Fig. 3:**
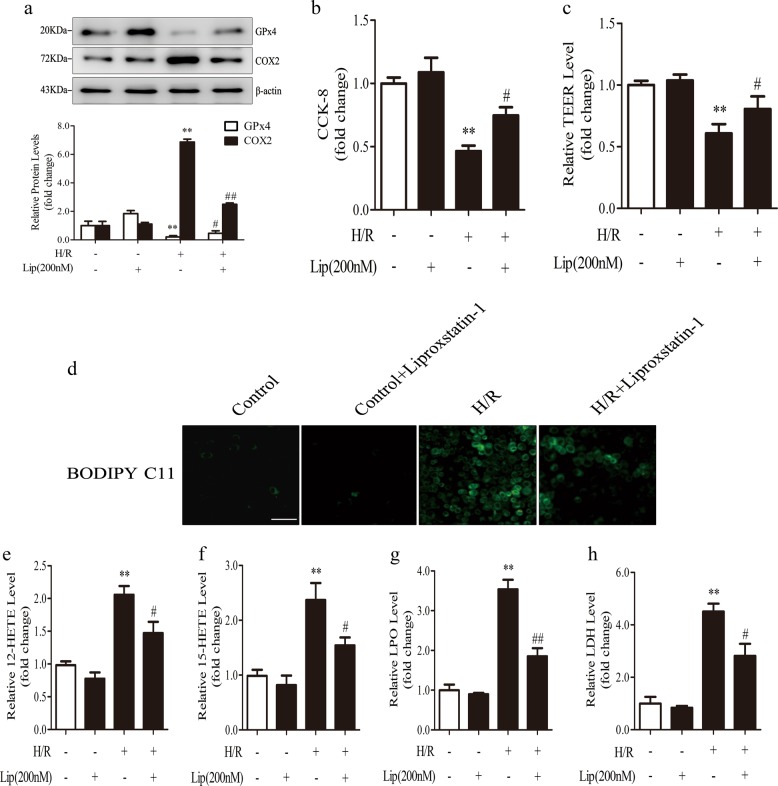
Liproxstatin-1 inhibits lipid peroxidation and cell death in vitro. Caco-2 cells were pretreated with liproxstatin-1 (200 nM) for 12 h before being subjected to H/R (H, 12 h of hypoxia; R, 2 h of reoxygenation). All samples were collected after H/R. **a** GPx4 and COX2 expression in cells were detected by western blotting (*n* = 3). **b** Cell survival was determined by CCK-8 kit (*n* = 6). **c** Transepithelial electrical resistance (TEER) level (*n* = 6). **d**–**g** Cell lipid peroxidation were detected by BODIPY 581/591 C11 staining using fluorescence microscopy (scale bar = 100 μm) and 12-HETE, 15-HETE, and LPO assay kits (*n* = 6). **h** The level of LDH (*n* = 6). All results are expressed as the mean ± SD. **p < 0.01 vs. the control group; #*p* < 0.05, ##*p* < 0.01 vs. the H/R group

In vitro, Caco-2 cells were treated with liproxstatin-1 for 12 h and then subjected to H/R. Consistent with the in vivo results, liproxstatin-1 effectively rescued GPx4 expression and suppressed COX2 expression (Fig. 3a). Liproxstatin-1 decreased cell death observed in the CCK-8 assay (Fig. 3b) and increased TEER (Fig. 3c). In addition, liproxstatin-1 decreased the density of green fluorescence represented by BODIPY 581/591 C11 staining after H/R (Fig. 3d) and reduced the levels of 12-HETE, 15-HETE, and LPO (Fig. 3e-g), suggesting that liproxstatin-1 prevents lipid peroxidation after H/R. LDH release was also decreased with liproxstatin-1 treatment (Fig. 3h).

Collectively, our findings confirm the involvement of ferroptosis in intestinal I/R injury and reveal that liproxstatin-1 protects against I/R injury and restores intestinal epithelial barrier function by inhibiting ferroptosis.

### Ferroptosis inhibition attenuates acute remote organ injury after intestinal I/R

**Fig. 4:**
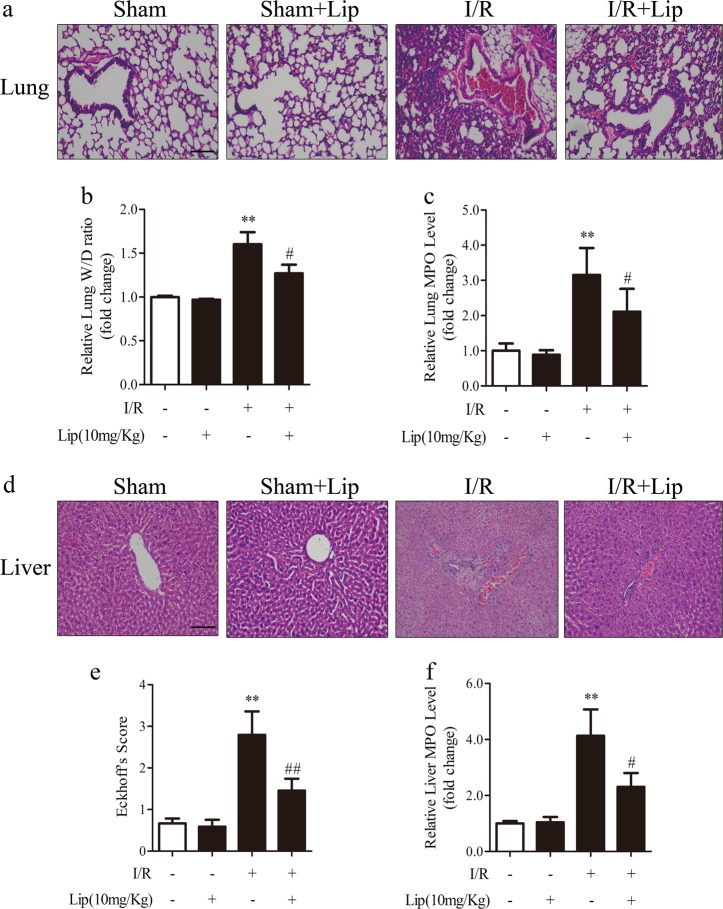
Liproxstatin-1 protects remote organs after intestinal I/R. Mice were treated with liproxstatin-1 (10 mg/kg) by intraperitoneal injection 1 h before ischemia and then subjected to ischemia/reperfusion (I, 45 min of ischemia; R, 30 min of reperfusion) or sham surgery (sham). All samples were collected after I/R. **a**,**d** Slices of remote organs (liver and lung) were stained by H&E and representative images were acquired by microscopy (scale bar = 100 μm). **b**,**c** Lung injury was measured by the wet/dry ratio and MPO assay kit (*n* = 6). **e**,**f** Liver injury was assessed by the Eckhoff’s score and MPO assay kit (*n* = 6). All results are expressed as the mean ± SD. **p < 0.01 vs. the sham group; #*p* < 0.05, ##*p* < 0.01 vs. the I/R group

Severe intestinal damage caused by I/R may lead to acute injury of remote organs such as the lung and liver, and this damage has a critical role in prognosis [39]. Thus, we examined the histopathological changes in the lung and liver after intestinal I/R. We found that ferroptosis inhibition by liproxstatin-1 mitigated histological injury of lung and liver (Fig. 4a and Fig. 4d, e). Liproxstatin-1 also significantly reduced lung edema (Fig. 4b) and decreased MPO activity in the lung and liver (Fig. 4c, f). These results indicate that ferroptosis inhibition prevents intestinal I/R-induced lung and liver injury.

### Inhibition of ischemia-induced ACSL4 suppresses lipid peroxidation and ferroptosis after reperfusion

**Fig. 5:**
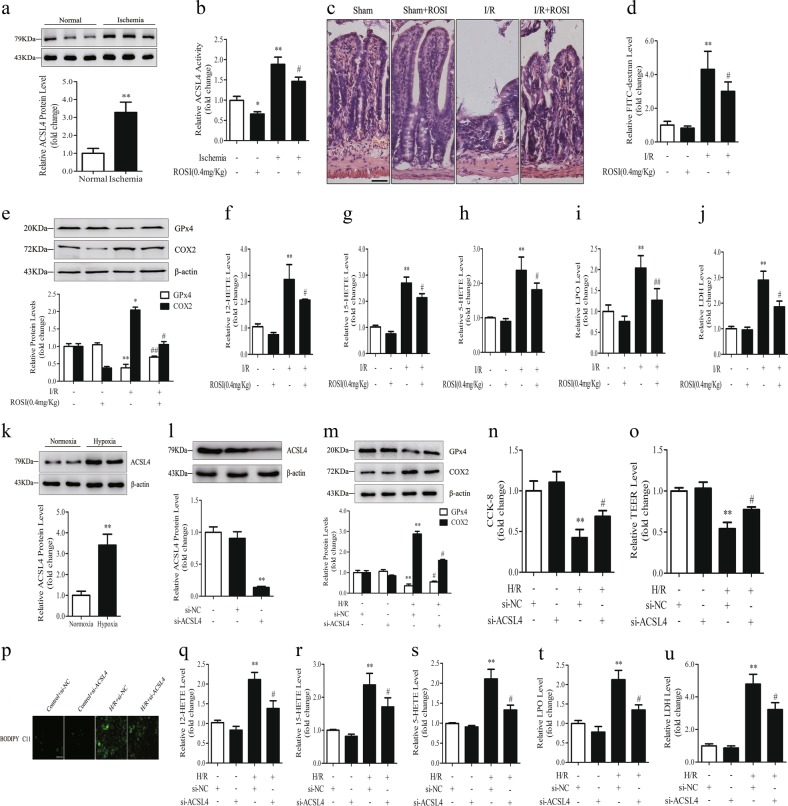
ACSL4 regulates ferroptosis in vivo and in vitro. **a** Western blotting was used to determine ACSL4 expression in normal and ischemic human intestines (*n* = 3). **p < 0.01 vs. the normal group. **b**–**j** ROSI (0.4 mg/kg, intravenous injection, 1 h before ischemia) was administered to mice. Then mice were subjected to ischemia, I/R, or sham surgery (I, 45 min of ischemia; R, 30 min of reperfusion). **b** ACSL4 activity in 45 min ischemic intestines (*n* = 6). *p < 0.05, **p < 0.01 vs. the sham group; #*p* < 0.05 vs. the ischemia group. **c** Representative H&E-stained images were acquired by microscopy after I/R (scale bar = 100 μm). **d** The intestinal permeability was detected by measuring serum FD-4 content after I/R (*n* = 6). **e** GPx4 and COX2 protein levels were assessed by western blotting after I/R (*n* = 3). **f**–**i** Lipid peroxidation was analyzed by 12-HETE, 15-HETE, 5-HETE, and LPO kits after I/R (*n* = 6). **j** Serum from mice was used to detect the level of LDH after I/R (*n* = 6). Results are expressed as the mean ± SD. *p < 0.05, **p < 0.01 vs. the sham group; #*p* < 0.05, ##*p* < 0.01 vs. the I/R group. **k**–**u** Caco-2 cells were transfected with si-NC or si-ACSL4 for 2 days before hypoxia or H/R (H, 12 h of hypoxia; R, 2 h of reoxygenation). **k**,**l** ACSL4 expression under hypoxia for 12 h or after siRNA transfection was assessed by western blotting (*n* = 3). **m** GPx4 and COX2 protein levels were determined by western blotting after H/R (*n* = 3). **n** Cell survival was measured by CCK-8 kit after H/R (*n* = 6). **o** Transepithelial electrical resistance (TEER) after H/R (*n* = 6). **p**–**t** Cell lipid peroxidation were detected by BODIPY 581/591 C11 staining using fluorescence microscopy (scale bar = 100 μm) and 12-HETE, 15-HETE, 5-HETE, and LPO assay kits after H/R (*n* = 6). **u** The level of released LDH after H/R (*n* = 6). Results are expressed as the mean ± SD. **p < 0.01 vs. the control group; #*p* < 0.05 vs. the H/R group

ACSL4 is a pivotal contributor and regulator of ferroptosis and it dictates ferroptosis sensitivity [21, 29]. However, whether ACSL4 can regulate ferroptosis-associated I/R injury is unknown. Thus, we assessed ACSL4 expression in ischemic intestinal tissues from humans and mice, and we found that ACSL4 was upregulated in ischemic tissues compared with that in normal tissues (Fig. 1a, Fig. 5a). Then, we established an ischemia model in mice pretreated with or without ROSI, which could inhibit ACSL4 and prevent ferroptosis [21]. After ROSI administration, ACSL4 was inhibited in the ischemia + ROSI group compared with in the ischemia group (Fig. 5b). Next, we established an I/R model in mice pretreated with or without ROSI. The H&E staining and serum FD-4 content results showed that ROSI protected against I/R injury and attenuated intestinal barrier dysfunction (Fig. 5c, d). Meanwhile, ROSI restored GPx4 expression and reduced COX2 expression (Fig. 5e). In addition, ROSI decreased the lipid peroxidation indicated by the reduced levels of 12-HETE, 15-HETE, 5-HETE, and LPO (Fig. 5f-i). LDH level was also decreased by ROSI (Fig. 5j). In addition, orally administrated ROSI in mice ameliorated intestinal I/R injury (Fig. S1A, C).

Then we transfected ACSL4 siRNA into Caco-2 cells to verify the function of ACSL4 inhibition in vitro. ACSL4 siRNA markedly decreased the hypoxia-induced expression of ACSL4 (Fig. 5k, l). Moreover, ACSL4 siRNA increased GPx4 expression and decreased COX2 expression (Fig. 5m), and rescued H/R-induced cell death determined by CCK-8 assay (Fig. 5n) and intestinal epithelial resistance (Fig. 5o). Furthermore, ACSL4 knockdown decreased lipid peroxidation as indicated by the decreased levels of green fluorescence (BODIPY 581/591 C11), 12-HETE, 15-HETE, 5-HETE, and LPO (Fig. 5p-t), and reduced the release of LDH (Fig. 5u).

In summary, the inhibition of ischemia-induced ACSL4 protected against ferroptosis and lipid peroxidation, and ameliorated the cell damage and intestinal barrier dysfunction caused by intestinal I/R.

### Sp1 regulates the mRNA and protein levels of ACSL4 in intestinal ischemia

**Fig. 6:**
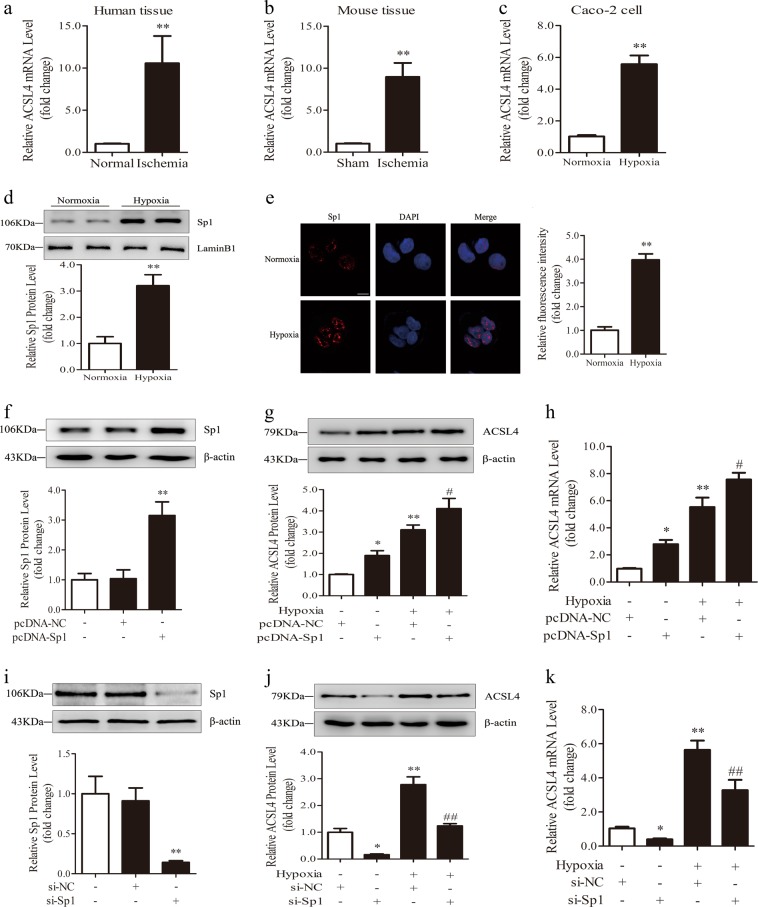
Sp1 regulates ACSL4 transcription and expression. **a**–**c** Ischemic human intestines, 45 min ischemic mouse intestines and 12 h hypoxic Caco-2 cells were used for the determination of ACSL4 mRNA levels by qPCR (*n* = 3). **p < 0.01 vs. the normal/sham/normoxia groups. **d**, **e** Expression of nuclear Sp1 at 12 h of hypoxia was determined by western blotting (*n* = 3) and laser scanning confocal microscopy (*n* = 6). **p < 0.01 vs. the normoxia group. **f**–**k** Caco-2 cells were transfected with Sp1 plasmid or siRNA for 2 days, and then subjected to 12 h of hypoxia. The Sp1 protein levels, the protein and mRNA levels of ACSL4 were determined after hypoxia *(n* = 3). Results are expressed as the mean ± SD. *p < 0.05, **p < 0.01 vs. the control group; #*p* < 0.05, ##*p* < 0.01 vs. the hypoxia group

According to a database prediction, the ACSL4 promoter region harbors abundant GC boxes. As a classical and vital transcription factor, Sp1 is characterized by a high affinity to GC-box promoter elements [30, 31]. Thus, we investigated whether Sp1 regulated the transcription of ACSL4. As shown in Fig. 6a and b, the expression of ACSL4 mRNA was increased in human and mouse intestinal tissues subjected to 45 min of ischemia and 12-hour hypoxia increased the ACSL4 mRNA level in Caco-2 cells (Fig. 6c). Using western blotting and immunofluorescence, we found a substantial increase in Sp1 expression in the nuclei of Caco-2 cells under hypoxic conditions (Fig. 6d, e). Furthermore, Sp1 overexpression increased the mRNA and protein levels of ACSL4 over the levels achieved by hypoxia alone (Fig. 6f-h). In contrast, Sp1 siRNA decreased the mRNA and protein levels of ACSL4 (Fig. 6i-k). These results indicate that Sp1 may regulate ACSL4 expression at the transcriptional level and induce ACSL4 expression after hypoxia.

In addition, inhibition of Sp1 in Caco-2 cells also decreased cell injury, lipid peroxidation, and COX2 expression caused by H/R (Fig. S2).

### Sp1 regulates ACSL4 transcription in Caco-2 cells by binding to the ACSL4 promoter region

**Fig. 7:**
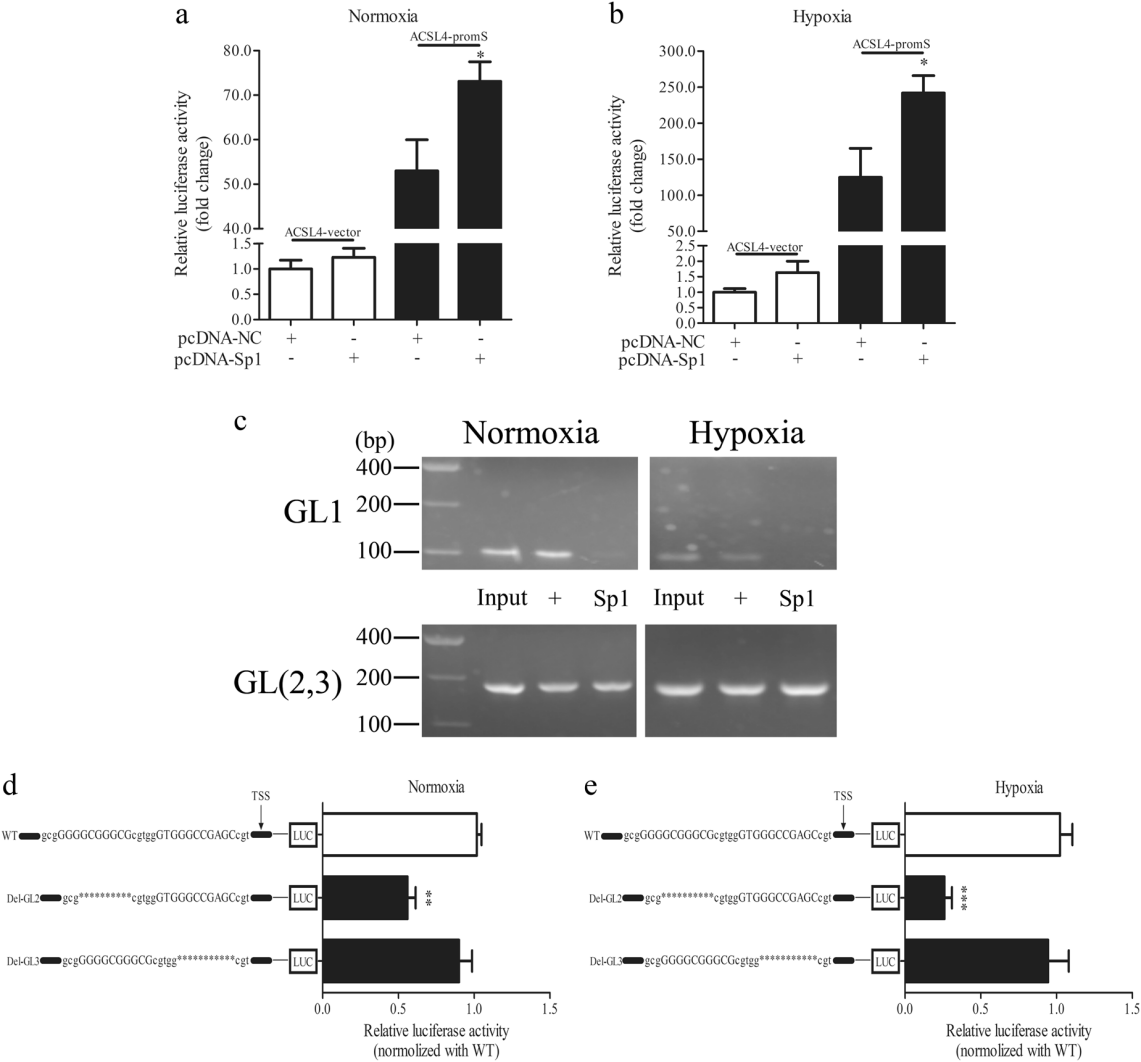
Sp1 binds the ACSL4 promoter region. Caco-2 cells were collected for assay with or without 12 h of hypoxia. **a**, **b** Cells cotransfected with the Sp1 and WT (− 500~ + 200) plasmids were collected and luciferase activity was analyzed (*n* = 3). *p < 0.05 vs. the empty vector group. **c** ChIP assay results for three sites were presented on agarose gels, grouped as input, positive and Sp1 (*n* = 3). **d**,**e** Cells transfected with two Del plasmids were used in the luciferase assay and the results were normalized to those of the WT group (*n* = 3). **p < 0.01, ***p < 0.001 vs. the WT group

We have proven that Sp1 promotes ACSL4 expression at the transcriptional level. Thus, we performed a database analysis to predict the binding motif(s) on the ACSL4 promoter region to verify the binding position. A segment was identified as a putative target (− 500~ + 200 bp relative to the transcription start site (TSS)). To determine the function of this region, we then generated a WT plasmid that contained the cloned − 500~ + 200 fragment fused to a luciferase reporter vector. The WT vector or empty vector was cotransfected with an Sp1/negative plasmid for the dual-luciferase reporter assay. Luciferase activity increased in the WT group compared with that in the empty vector group in both normoxic and hypoxic conditions (Fig. 7a, b). To confirm the exact position at which Sp1 could bind within this fragment, we assessed the interactions with the three GC-box-like motifs (GL1, GL2, and GL3) listed in Table 1 using a ChIP assay. As shown in Fig. 7c, in both normoxic and hypoxic conditions, Sp1 bound to the region of the ACSL4 promoter containing GL2 and GL3 but not GL1. Then, the Del plasmids were used to further clarify the function of GL2 and GL3. The results of the dual-luciferase reporter assay indicated that GL2 (GGGGCGGGCG) was a functional site controlling ACSL4 transcription (Fig. 7d, e).

**Table 1:** Potential Sp1-interacting motifs in the 5′-upstream flanking region of the *ACSL4* gene

Location (relative to TSS)	Sequence (5′ to 3′)	Orientation
GL1 (from − 286 to − 277)	GGGGTGGAGT	F
GL2 (from − 38 to − 29)	GGGGCGGGCG	F
GL3 (from − 23 to − 13)	GTGGGCCGAGC	R

Table 1 Three potential Sp1-interacting motifs were identified within the region 500 bp upstream of the ACSL4 promoter based on the consensus GC-box sequence. The orientation (R; reverse, F; forward) and the location of each motif relative to the TSS are shown

These results suggest that Sp1 directly activates *ACSL4* gene transcription by binding to a specific locus on the ACSL4 promoter under both normoxic and hypoxic conditions.

## Discussion

As ferroptosis was identified by Stockwell et al. [12, 40, 41] as a new form of RCD, it has been closely correlated with human diseases. Four key factors, namely iron, PUFAs, oxygen, and a reduction in antioxidants, are indispensable for ferroptosis induction [17]. Thus, we hypothesized that ischemia or hypoxia may be an important process facilitating the occurrence of ferroptosis during reoxygenation. To validate this hypothesis, in the present study, we examined the expression levels of core proteins, iron, GPx4 activity, and the content of GSH in ischemic intestinal tissues from mice. We found the proferroptotic factors increased and the antiferroptotic factors decreased, thus indicating that the epithelium might experience ferroptosis during reperfusion or reoxygenation. Furthermore, we established an ischemia model incorporating various reperfusion durations and examined the salient features of ferroptosis in situ. The TEM and western blotting results showed that ferroptosis occurred after 30 min of reperfusion, which is a relatively early phase. Next, with the use of liproxstatin-1, which can specifically inhibit ferroptosis, we determined whether ferroptosis inhibition could rescue intestinal I/R injury. Our findings indicated that liproxstatin-1 ameliorated histological injury to the intestine, increased intestinal cell viability, restored epithelial barrier function, inhibited lipid peroxidation, and decreased the release of detrimental factors. Thus, we conclude that ferroptosis occurs at the early phase of reperfusion, thus leading to intestinal injury, and that the inhibition of ferroptosis protects against this lethal process.

As a pivotal indicator and regulator of ferroptosis, ACSL4 functions as a critical determinant of ferroptosis sensitivity by modulating the cellular (phospho)lipid composition. ACSL4-knockout cells were resistant to lipid peroxidation and ferroptosis [21]. However, ACSL4 has not been investigated in I/R injury associated with ferroptosis. In our study, we found that ACSL4 expression was upregulated under ischemic conditions and contributed to reperfusion-induced ferroptotic injury. To suppress ACSL4, we pretreated ROSI in mice or siRNA in Caco-2 cells, to achieve pharmacological or genetic inhibition of ACSL4 under ischemic or hypoxic conditions. The results indicated that the inhibition of ACSL4 before reperfusion or reoxygenation can rescue cells from injury by abolishing ferroptosis. In a further study, we aimed to determine the regulator that induces ACSL4 expression. After a database analysis, we found a potential candidate regulator, namely Sp1, which is a classical transcription factor that binds to GC-box motifs in target gene promoters. We proved that Sp1 could regulate ACSL4 expression and validated an effective binding site (located at − 38~ − 29 bp relative to the TSS).

The transient induction of GPx4 at 30 min of ischemia observed in this study was thought to be involved in a possible form of transient profit under a period of hypoxia [42]. However, a continuous decrease in GPx4 expression was found in prolonged ischemia, which was responsible for reperfusion-induced ferroptosis, as GPx4 inhibition invariably causes ferroptosis [43].

In addition to decreased GPx4 protein expression, GPx4 activity, GSH level, and GSH/GSSG ratio were significantly decreased after 45 min of intestinal ischemia. GPx4 activity, which is critical for ferroptosis execution [44, 45], could be affected by diverse factors [46–48], such as GSH, selenium, etc. Further studies are needed to determine how these factors are involved in intestinal ischemia.

Apoptosis [49], necroptosis [50], and autophagy [51] have been demonstrated to be involved in intestinal I/R injury, but ferroptosis has not been studied. Our study indicated that ferroptosis has an important role in intestinal I/R injury and could be a lethal process induced by reperfusion. As a dynamic process, I/R undeniably involves complex cell death mechanisms. However, the orchestrated connections among these cell death processes need to be studied in greater depth.

Here we found that ferroptosis occurred in the early phase of reperfusion. Interestingly, in previous reports, apoptosis was found to occur at a substantially later phase [52–54]. Thus, we were prompted to consider whether ferroptosis and apoptosis were intimately related in the I/R model. Dixon indicated that apoptosis can be recognized as a form of “suicide”, whereas ferroptosis is termed “sabotage”. Although ferroptosis and apoptosis involve different prodeath proteins and mechanisms, which may lead to the separation of cell sabotage from cell suicide [55], some studies have shown the potential connection between ferroptosis and apoptosis. Lee et al. [56, 57] reported that the p53-independent CHOP/PUMA axis responds to ferroptosis inducers; this response may have a key role in the ferroptotic agent-mediated sensitization to TRAIL-induced apoptosis. In addition, vitamin E, which is an antioxidant that has been used to inhibit apoptosis in I/R models, can fully prevent ferroptotic cell death [12]. In our model, reperfusion-induced ferroptosis produced abundant L-ROS and destroyed the cellular structure. These changes may partially create the precondition for apoptosis.

In addition, necroinflammation has been found to be involved in primary and distant organ injury following acute kidney injury [58, 59]. However, the role of necroinflammation in intestinal I/R, especially links with ferroptosis, remains unexplored. It has been proven that ferroptotic cells trigger the innate immune system in mouse models of the brain and kidney disease [60]. The activated innate immune system can initiate local inflammation and subsequently lead to necroinflammation in remote organs via spreading damage-associated molecular patterns and cytokines to systemic circulation from primary ischemic organ [59, 61]. In the present study, I/R-induced ferroptosis likely activated the innate immune system and led to the infiltration of immune cells [62] in the intestine. Furthermore, the activated immune system led to the release of cytokines, which may ultimately cause necroinflammation in the intestine, liver, and lung. It is an important and promising mechanism that needs to be further studied.

In addition to the crosstalk between ferroptosis and other forms of cell death, the function and assay of ferroptosis-related molecules needs to be investigated. Some newly reported proteins such as PEBP1 [63], NCOA4 [64, 65], and metallothionein-1G [66] are correlated with ferroptosis via iron metabolism and lipid peroxidation. Further studies on ferroptosis are needed not only to clarify the molecular mechanism but also to provide an opportunity for designing new therapeutic interventions. For example, in the treatment of advanced hepatocellular carcinoma, sorafenib resistance has been shown to result from the metallothionein-1G-induced inhibition of ferroptosis [66]. This discovery indicates promise for the incorporation of ferroptosis into clinical treatment. For the ferroptosis assay, we assayed lipid peroxidation (HETEs, LPO, lipid-ROS dye), combined with iron, TEM, and related protein expression, as in previous studies [9, 38, 67]. In a recent breakthrough, studies have indicated that oxidative lipidomics is currently the best established method to detect lipid peroxidation, specifically peroxidation of PE, which is a hallmark of ferroptosis [18, 22, 68].

In conclusion, our study reveals the function of ferroptosis in intestinal I/R injury and demonstrates that the inhibition of ferroptosis can ameliorate in situ and remote organ injury. ACSL4, a protein vital to ferroptosis, is induced after ischemia and is involved in I/R injury. Sp1 can upregulate ACSL4 expression by binding to the ACSL4 promoter region. These findings guide us to extend ferroptosis inhibition to intestinal I/R treatment.

## Supplementary information

Supplementary Figure S1

Supplementary Figure S2

Supplemental figure legends
