# Supplementary figures and images for: Ischemia-induced ACSL4 activation contributes to ferroptosis-mediated tissue injury in intestinal ischemia/reperfusion
Source: Cell Death Differ. 2019 Feb 8;26(11):2284–99. doi: 10.1038/s41418-019-0299-4 (PMC6889315; doi:10.1038/s41418-019-0299-4)

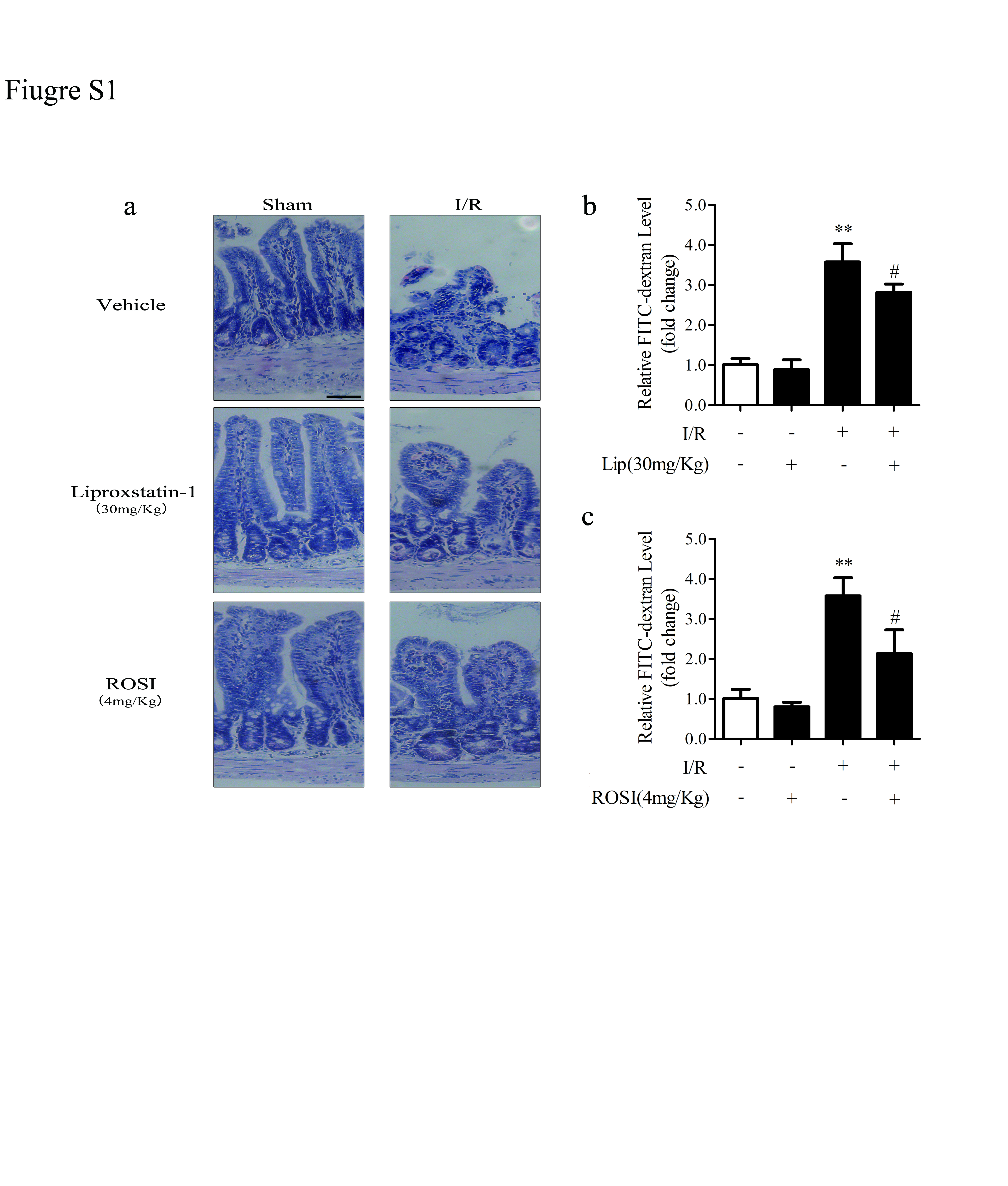

Supplement: Supplementary file 1 — Supplementary Figure S1 [file 41418_2019_299_MOESM1_ESM.tif]

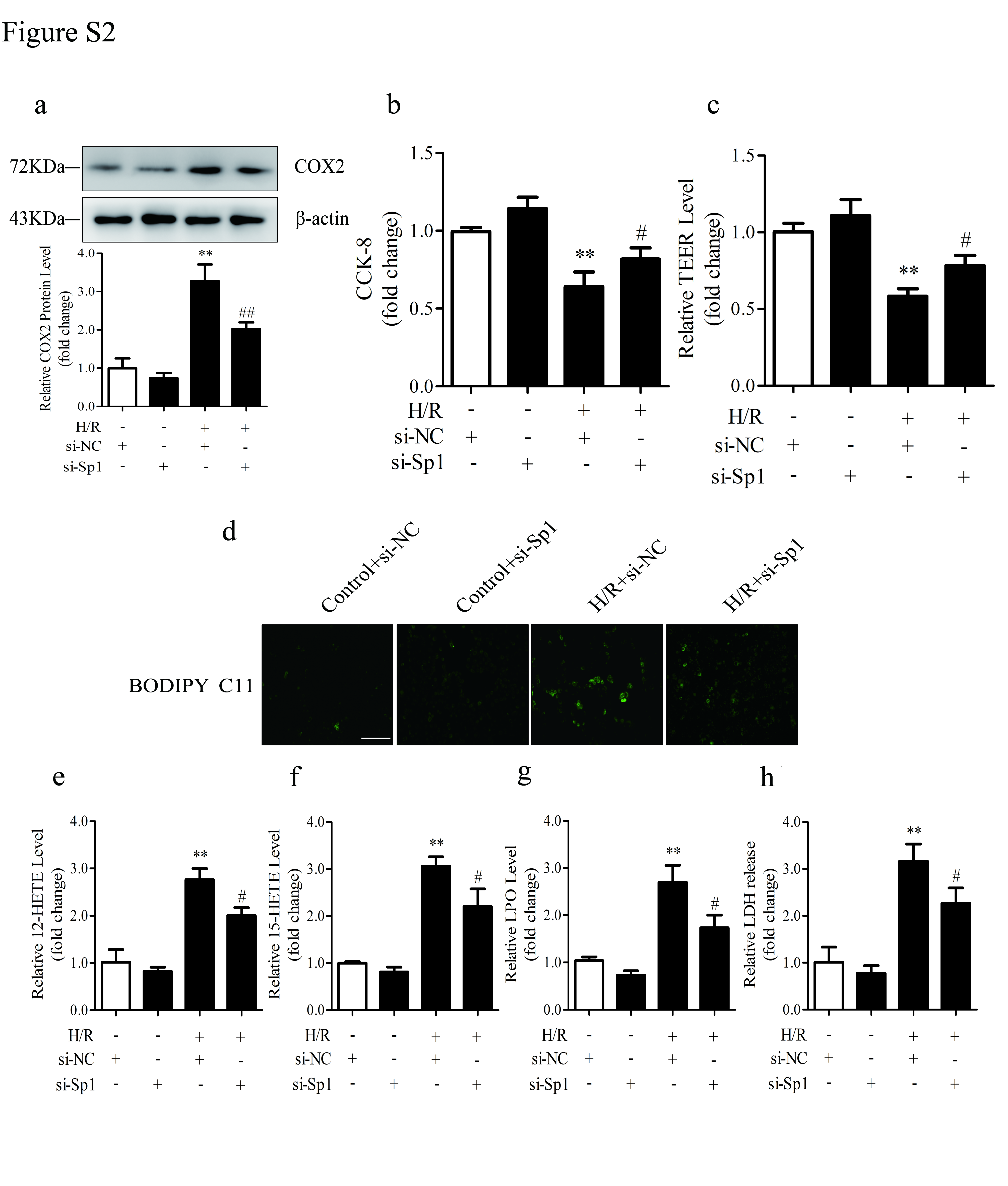

Supplement: Supplementary file 2 — Supplementary Figure S2 [file 41418_2019_299_MOESM2_ESM.tif]
