# Supplementary material for: Ischemia-induced ACSL4 activation contributes to ferroptosis-mediated tissue injury in intestinal ischemia/reperfusion
Source: Cell Death Differ. 2019 Feb 8;26(11):2284–99. doi: 10.1038/s41418-019-0299-4 (PMC6889315; doi:10.1038/s41418-019-0299-4)
Supplement: Supplementary file 3 — Supplemental figure legends [file 41418_2019_299_MOESM3_ESM.docx]

**Legends**

**Fig. S1 Oral administration of liproxstatin-1 and ROSI ameliorates intestinal I/R injury.** Mice were treated with liproxstatin-1 (30 mg/kg) and ROSI (4 mg/kg) for 3 days (once per day) by intragastric administration (*i.g.*) before I/R (I, 45 min of ischemia; R, 30 min of reperfusion). **A** Representative H&E-stained intestinal slices after I/R were imaged by microscopy (scale bar=100μm). **B, C** Intestinal permeability was detected by measuring serum FD-4 content after I/R (n=6). ***p*<0.01 versus the sham group; #*p*<0.05 versus the I/R group.

**Fig. S2 Inhibition of Sp1 decreases H/R mediated lipid peroxidation and cell injury *in vitro*.** Caco-2 cells were transfected with si-Sp1 or si-NC for 2 days before H/R (H, 12 hours of hypoxia; R, 2 hours of reoxygenation). All samples were collected after H/R. **A** The expression of COX2 was assayed by western blotting (n=3). **B** Cell survival was measured by CCK-8 kit (n=6). **C** Transepithelial electrical resistance (TEER) (n=6). **D-G** Cell lipid peroxidation were detected by BODIPY 581/591 C11 staining using fluorescence microscopy (scale bar=100μm) and 12-HETE, 15-HETE, and LPO assay kits (n=6). **H** The level of released LDH (n=6). All results are expressed as the mean±SD. ***p*<0.01 versus the control group; #*p*<0.05 versus the H/R group.
